# *QuickStats:* Percentage* of Adults Aged ≥18 Years with Arthritis,^†^ by Sex and Age Group — National Health Interview Survey,^§^ United States, 2019

**DOI:** 10.15585/mmwr.mm7017a7

**Published:** 2021-04-30

**Figure:**
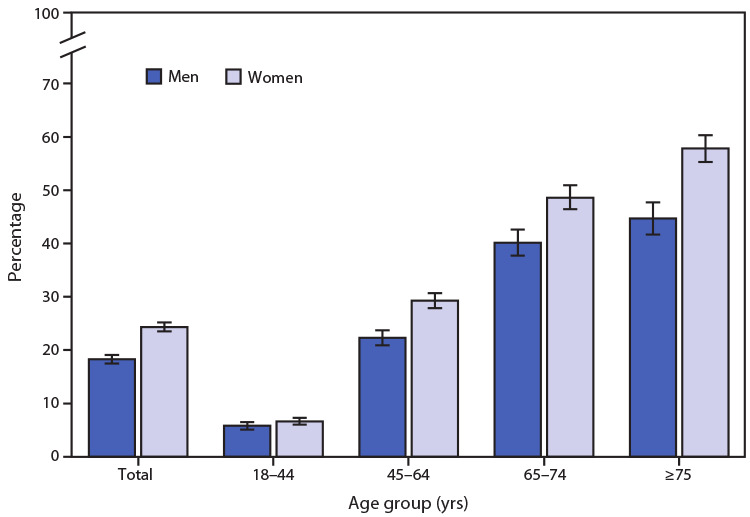
In 2019, among adults aged ≥18 years, prevalence of arthritis (including rheumatoid arthritis, gout, lupus, and fibromyalgia) increased with age among both men and women. For men, prevalence increased from 5.8% among those aged 18–44 years to 22.3% among those aged 45–64 years, 40.1% among those aged 65–74 years, and 44.7% among those aged ≥75 years. For women, prevalence increased from 6.6% among those aged 18–44 years to 29.3% among those aged 45–64 years, 48.6% among those aged 65–74 years, and 57.8% among those aged ≥75 years. Women were more likely to have arthritis than were men overall (24.3% versus 18.3%) and in all age groups except 18–44 years, where the difference did not reach statistical significance.

**Source:** National Center for Health Statistics. National Health Interview Survey, 2019. https://www.cdc.gov/nchs/nhis.htm

